# Self-confidence in the management of health complications at school: contributions of the *in situ* simulation^*^

**DOI:** 10.1590/1518-8345.2909.3174

**Published:** 2019-10-07

**Authors:** Jaqueline Brosso Zonta, Aline Helena Appoloni Eduardo, Maria Verônica Ferrareze Ferreira, Gabriela Heleno Chaves, Aline Cristiane Cavicchioli Okido

**Affiliations:** 1 Universidade Federal de São Carlos, São Carlos, SP, Brasil.; 2 Universidade Federal de São Carlos, Departamento de Enfermagem, São Carlos, SP, Brasil.; 3 Universidade de São Paulo, Escola de Enfermagem de Ribeirão Preto, Centro Colaborador da OPAS/OMS para o Desenvolvimento da Pesquisa em Enfermagem, Ribeirão Preto, SP, Brasil.

**Keywords:** Child, School Health Services, Nursing, First Aid, Simulation, Trust, Criança, Serviços de Saúde Escolar, Enfermagem, Primeiros Socorros, Simulação, Confiança, Niño, Servicios de Salud Escolar, Enfermería, Primeros Auxilios, Simulación, Confianza

## Abstract

**Objective:**

to analyze the contributions of the *in situ* simulation in the self-confidence of early childhood and elementary education teachers regarding the initial management of health complications in school.

**Method:**

this is a pre-post testing quasi-experimental study. Two pre and post *in situ* simulation instruments were applied to 76 teachers, namely: visual analogue scale of teachers’ self-confidence in the management of health complications at school, and a questionnaire to assess their knowledge on the subject. The educational activity was composed of four scenarios of *in situ* simulation. The data were analyzed by descriptive and analytical statistics using univariate and multivariate linear regression.

**Results:**

the comparison of results of pre and post *in situ* simulation self-confidence identified promotion of self-confidence (p<0.001), especially for those teachers with less professional experience (p=0.008), without previous similar experience (p=0.003) and who actively participated in the simulation (p=0.009).

**Conclusion:**

the teachers feel uncomfortable to handle health complications. The *in situ* simulation elevated the perception of self-confidence among teachers.

## Abstract (pt)

**Objetivo:**

analisar as contribuições da simulação *in situ* na autoconfiança de professores da educação infantil e fundamental I com relação ao manejo inicial das intercorrências de saúde na escola.

**Método:**

estudo quase experimental, do tipo pré e pós-teste. Aplicou-se em 76 professores dois instrumentos pré e pós simulação *in situ*, os quais foram: escala visual analógica de autoconfiança dos professores para manejo das intercorrências de saúde na escola e questionário para avaliar o conhecimento na temática. A atividade educativa foi composta por quatro cenários de simulação *in situ*. Os dados foram analisados mediante estatística descritiva e analítica, utilizou-se regressão linear univariada e multivariada.

**Resultados:**

a comparação dos resultados de autoconfiança pré e pós simulação *in situ* identificou promoção da autoconfiança (p<0,001) em especial para aqueles professores com menor tempo de experiência profissional (p=0.008), sem vivência prévia semelhante (p=0.003) e que participaram ativamente da simulação (p=0.009).

**Conclusão:**

os professores sentem-se pouco confiantes para manejar intercorrências de saúde. A simulação *in situ* elevou a percepção da autoconfiança entre os professores.

## Abstract (es)

**Objetivo:**

analizar las contribuciones de la simulación *in situ* en la autoconfianza de profesores de la educación infantil y primaria respecto al manejo inicial de las intercurrencias de salud en la escuela.

**Método:**

estudio cuasiexperimental, del tipo pre- y posprueba. Se aplicaron a 76 profesores dos instrumentos pre- y postsimulación *in situ*, a saber: escala visual analógica de autoconfianza de los profesores para manejo de las intercurrencias de salud en la escuela y cuestionario para evaluar el conocimiento sobre el tema. La actividad educativa comprendió cuatro escenarios de simulación *in situ*. Los datos se analizaron mediante estadística descriptiva y analítica, se empleó la regresión lineal univariada y multivariada.

**Resultados:**

la comparación de los resultados de autoconfianza pre- y postsimulación *in situ* identificó la promoción de la autoconfianza (p<0,001) en especial en aquellos profesores con menor tiempo de experiencia profesional (p=0,008), sin vivencia previa semejante (p=0,003) y que participaron activamente en la simulación (p=0,009).

**Conclusión:**

los profesores se sienten poco confiados para manejar intercurrencias de salud. La simulación *in situ* aumentó la percepción de la autoconfianza entre los profesores.

## Introduction

During childhood, a sharp immune and neurological development occurs, which makes the child vulnerable to diseases and accidents, as well as more susceptible to complications and death^1^. Health complications in childhood can be subdivided between the ones related to diseases, such as respiratory and gastrointestinal disorders, and the ones related to accidents, such as traffic accidents, poisoning, drowning, burns, falls and airway obstruction^1-2^.

Most complications related to health in childhood happen at home. However, the school environment is not free of these complications, since children stay a large part of the day there^3-7^. Thus, actions of prevention and health promotion are required in schools and, to this end, it is essential that teachers and other professionals working in this environment be able to handle first aid^5-9^.

First aid in the school environment is generally conducted by teachers^10-11^; however, they have low levels of knowledge on the subject^10-12^. They report not having any kind of continuous or systematic training and that, when necessary, they use knowledge obtained in readings and/or previous experiences, in addition to common sense^13^. Literature reinforces that negative feelings such as insecurity, fear and nervousness are enhanced in the face of health complications in the school context, weakening teachers’ self-confidence^14-15^. In this perspective, this investigation shares the assumption that self-confidence, combined with previous experiences and knowledge may promote a safe management of health complications^16-17^.

Literature shows the use of several teaching strategies that aim to promote skill and knowledge on the management of health complications among teachers, such as expository classroom, educational booklet and videos^7,18^. However, no studies that adopted the simulation strategy among teachers were accessed, being common among health professionals^16,19-21^.

Among the approach possibilities using simulations, this study adopted the *in situ* simulation. *In situ* simulation is every activity based on simulation that occurs in the real context, that is, the simulated scenarios are built in the working environment itself^22-23^. It facilitates professionals’ access to training and enhances the training of real professional teams^23^. In addition, it promotes the fidelity of the scenarios as the learning context is similar to the context of practice^22^.

Therefore, a study demonstrates that the *in situ* simulation may increase the levels of confidence, resulting in improved recognition and management of situations^24^. Thus, this investigation innovates by proposing an educational intervention mediated by the *in situ* simulation among early childhood and elementary education teachers.

Faced with the exposed, the following research questions were established: does an educational activity mediated by *in situ* simulation promote self-confidence in relation to the management of health complications in the school? What are the factors associated with the promotion of self-confidence after an educational activity mediated by *in situ* simulation?

Therefore, the objective of this study was to analyze the contributions of *in situ* simulation in the self-confidence of early childhood and elementary education teachers regarding the initial management of health complications in school.

## Method

Pre-post testing quasi experimental study^25^, developed in four public institutions of early childhood and elementary education of a municipality from the interior of the state of São Paulo, from May to October 2017.

Regarding the study participants, the inclusion criteria were: being an early childhood or elementary education teacher, being 18 years-old or older, and having at least three months of professional experience. Those who were on vacation or leave, and teachers who were not present at all scheduled meetings were excluded. Thus, initially, 113 teachers agreed to participate in the study; however, 37 did not attend all the meetings and were excluded, and 76 teachers remained, with an average of 19 per school. No one refused.

The production of the empirical material began with the identification of public early childhood education and elementary schools of the municipality. Then, at random, the principals were contacted in order to explain about the research and to request permission to use two hours of collective pedagogical work (HCPW). It is important to state that the Municipal Department of Education previously authorized the study and that the researchers did not have any previous relation with the chosen institutions.

In the four schools included in the study, three meetings were scheduled, according to availability. In the first meeting, the eligible teachers who accepted the invitation answered the research instruments, which were: instrument for characterization of the teachers; visual analogue scale (VAS) of teachers’ self-confidence in the management of health complications in the school; and an assessment questionnaire on the knowledge to act before health complications in schools.

The VAS of teachers’ self-confidence to manage health complications in school is composed of 12 items that address the teachers’ perception of confidence in assessing and ensuring the safety of the place where the complication occurs, evaluating and verifying the need to call for help and in evaluating and offering first aid in the following situations: fever, choking, seizure, fall with deep wound and bleeding, and cardiorespiratory arrest. Each item of the scale has a horizontal line of 10cm with descriptors in the edges: “not confident at all” on the left and “completely confident” on the right. To establish the score, the respondent indicates along the line the level of self-confidence they have at the moment. The interpretation of results is given by measuring the space obtained between the extremity on the left and the point signaled by the respondent, with a ruler graduated in centimeters. The scale allows to access the level of confidence for each item, and also to measure the general average of self-confidence^26^.

The questionnaire to assess the knowledge to act in the face of health complications was prepared by the researchers and based on the analysis of national and international literature and on discussions between the members of the research group. Then, the content was validated by 11 expert judges. It has 42 items relative to the initial care of a student in situation of health complication, such as seizures, cardiopulmonary arrest, drowning, among others. For each item, there are three answer possibilities: true (1), false (0) and I do not know (0). In this way, the minimum score of the questionnaire is zero, and the maximum 42.

Then, a booklet was provided by e-mail, named “First aid in the school: guidelines for teachers of preschool early childhood and elementary education”^7^. The reading of the booklet is a stage of the simulation named “pre-briefing”, that is, every orientation, task or activity designed by the educator that occurs in a moment previous to the development of the scenario^27-28^. This stage aimed to bring the participants close to the theme in order to enhance performance in the simulated scenario and in the subsequent discussion. Theoretical classes, videos and readings are among the strategies commonly adopted in the pre-briefing^27^. It is important to record that, because it is an individual reading outside the time reserved for data collection and, therefore, was difficult to be controlled, this activity was not considered as a participation criterion in the research. However, when questioned, in general, teachers claimed to have carried out the preliminary reading.

The implementation of the educational activity medicated by the *in situ* simulation happened in the second and third meeting from four simulated scenarios developed in the school. The scenarios were elaborated from a methodological referential that comprises the following steps: identification of the theme, objective of the simulation, participants, pre-briefing, scenario and debriefing process^28^. The construction of scenarios was based on the current scientific evidence on the subject, as well as on the previous experience of the researchers. The four templates were subjected to validation of appearance and content by 11 expert judges. In addition, the scenarios were subjected to a pilot test with undergraduate students of the course of Pedagogy and Special Education during an internal university event.

The general objective of the educational activity mediated by the simulation *in situ* was to provide the learning of initial management of four health complications that may occur at school, which were: airway obstruction by foreign body/choking, fall with deep wound/bleeding, seizures and cardiopulmonary arrest. The active participation in scenarios was voluntary; however, all were encouraged to actively experiment at least one scenario. For each situation, two teachers participated actively, that is, were placed in the scenario to perform the care, and the others remained as observers. Each scenario was developed once in each school, therefore, 32 teachers participated and 44 observed. In the last meeting, after completing the educational activity, teachers answered the same instruments previously applied, except for the characterization.

Data were coded and included in a formatted database in the spreadsheet editor Excel, through double typing. After validation, the database was exported to the software Statistical Package for Social Science (SPSS), version 9.2, in which the statistical analyses were processed. As response variables, the average self-confidence score after *in situ* simulation and the difference between the self-confidence scores pre and post *in situ* simulation, named “self-confidence promotion”. The independent variables were: sex (categorical variable), age (categorical and/or numerical variable), education (categorical variable), children (dichotomous and/or numeric variable), professional experience in years (numerical variable); active participation (dichotomous variable); previous experience with health complication in school or in another environment, named “previous experience” (dichotomous variable) and knowledge promotion (numeric variable obtained from the difference between the knowledge scores pre and post *in situ* simulation).

In the descriptive statistic analysis, the categorical variables were shown as absolute and relative frequency, and the numeric as measures of central tendency, variability, and position. The Wilcoxon test was used for related samples to compare the mean self-confidence scores pre and post *in situ* simulation. Mann-Whitney test was used for dichotomous variables to compare the response variables between the independent variables, and the Kruskal-Wallis test for those with more than one category. According to Shapiro-Wilk and Kolmogorov-Smirnov tests of normality, there was no normal distribution of variables.

The correlation of numerical variables was calculated from Spearman’s correlation coefficient. A significance level of 5% was adopted for the tests^29^. Finally, to analyze the factors related to self-confidence, the univariate and multivariate linear regression analysis, with Stepwise selection criterion was used.

It is worth mentioning that the internal consistency of instruments used in the data collection was verified by the Cronbach’s alpha coefficient, considering values above 0.70 as high consistency(29). Thus, the value of Cronbach’s α of the VAS of teachers’ self-confidence to manage health complications in school and of the questionnaire to assess knowledge were 0.94 and 0.81, respectively.

It is important to record that the educational activity mediated by the *in situ* simulation had the participation of four undergraduate students, two graduate students and two teachers, and was linked to an extension activity. However, the application of instruments was conducted exclusively by the main researcher.

The project was approved by the Research Ethics Committee of the Federal University of São Carlos, under CAEE:65118117.9.0000.5504. All teachers received a copy of the Informed Consent Form (ICF), which informed on the objectives of the study, the data collection procedures, possible risks/embarrassment, benefits, as well as the guarantee of confidentiality and the respect to the desire to participate or not in the research.

## Results

A total of 76 early childhood and elementary education teachers participated in the study. Most were female (97%), with average of 39.4 years of age and of 12.6 years of professional experience.

The average self-confidence score pre *in situ* simulation was 4.13, standard deviation ±1.57, minimum score of 1.20, median 3.81 and maximum score of 9.11. After the *in situ* simulation, the mean self-confidence score was 6.92, standard deviation ±1.84, minimum score of 2.07, median of 6.78 and maximum score of 9.78. The difference of self-confidence averages between pre and post simulation, from the Wilcoxon test for related samples, revealed *p*<0.001.

There was an increase in the mean self-confidence score of 3.03. The item that showed the highest difference between the mean pre and post means (5.18) was: “I feel confident to offer first aid to a child that is having a seizure.” And the item with less difference (0.03) was: “I feel confident to recognize when a child has a fever.”

Table 1 shows the pre and post *in situ* simulation self-confidence scores for each one of the 12 items of the score, as well as the self-confidence promotion.

**Table 1:** – Teachers’ self-confidence to perform in situations of health complications in the school before and after *in situ* simulation. São Carlos, SP, Brazil, 2017

Item	Pre-simulation self-confidence	Post-simulation self-confidence	Self-confidence promotion
1. I feel confident to assess the safety of the place when a child shows a health complication, either due to illness or accident.	3.64	6.09	2.45
2. I feel confident to identify the need to call the Emergency Medical Service (SAMU) at phone number 192.	6.15	8.26	2.11
3. I feel confident to recognize when a child is having a seizure.	3.88	7.52	3.64
4. I feel very confident to offer first aid to a child who is having a seizure.	1.88	7.06	5.18
5. I feel confident to offer first aid to a child that fell.	3.75	6.53	2.78
6. I feel very confident to offer first aid to a child who suffered a wound that is bleeding too much.	3.68	6.39	2.71
7. I feel confident to recognize when a child is unconscious and not breathing.	3.27	6.08	2.81
8. I feel confident to offer first aid to an unconscious child who is not breathing.	1.35	5.53	4.18
9. I feel very confident to recognize when a child is choking.	4.56	6.93	2.37
10. I feel confident to offer first aid to a child that is choking.	2.61	6.42	3.81
11. I feel confident to recognize when a child has a fever.	8.19	8.16	0.03
12. I feel confident to offer first aid to a child that has a fever.	6.72	8.09	1.37

The mean scores of self-confidence post *in situ* simulation and of self-confidence promotion according to the categorical variables are shown in Table 2.

**Table 2:** – Distribution of the mean score of self-confidence post *in situ* simulation and of the mean score of self-confidence promotion according to the categorical variables. São Carlos, SP, Brazil, 2017

Variables	N (%)	Post-simulation self-confidence	*p*	Self-confidence promotion	*p*
Sex			0.844*		0.617*
Female	74 (97.4)	6.91		2.85	
Male	2 (2.6)	7.32		3.61	
Age			0.077^†^		0.289^†^
< 40 years	34 (47.2)	7.41		3.32	
40-49 years	25 (34.7)	6.50		2.84	
≥ 50 years	13 (18.1)	6.63		2.42	
No reply	4			-	
Education			0.304*		0.610*
Higher education	27 (36.5)	7.32		3.05	
Graduate studies	47 (63.5)	6.64		2.76	
No reply	2			-	
Professional experience			**0.001** ^†^		**0.011** ^†^
<10 years	33 (44.6)	7.83		3.31	
10-19 years	26 (35.1)	6.59		3.12	
≥20 years	15 (20.3)	5.69		1.67	
No reply	2			-	
Children			0.920*		0.819*
No	22 (29.3)	6.82		2.83	
Yes	53 (70.7)	6.92		2.95	
No reply	1			-	
Previous experience			0.131*		0.002*
No	16 (21.3)	7.59		4.10	
Yes	59 (78.7)	6.70		2.50	
No reply	1			-	
Active participation			0.009*		0.375*
No	44 (57.9)	6.53		2.71	
Yes	32 (42.1)	7.48		3.09	

*Mann-Whitney test; ^†^Kruskal-Wallis

The correlation matrix involving numeric variables and response variables is shown in Table 3.

**Table 3:** – Spearman’s correlation coefficient between response variables and numeric variables. São Carlos, SP, Brazil, 2017

Variables	Age	Professional experience	Number of children	Knowledge promotion
Mean score of self-confidence after educational activity	-0.258*	-0.378*	-0.132	-0.083
Mean score of self-confidence promotion	-0.202	-0.187	0.007	0.148

*p<0.005

The univariate linear regression analysis was used in this study to assess the relation of independent variables with mean score of self-confidence *in situ* simulation and with self-confidence promotion, as shown in Table 4.

**Table 4:** – Effect of the independent variables in the mean score of self-confidence post *in situ* simulation and in the self-confidence promotion, following a univariate linear regression model. São Carlos, SP, Brazil, 2017

Variable	Post-simulation self-confidence			Self-confidence promotion		

	Beta* (EP)^**†**^	*p*-value	R^**2‡**^	Beta* (SE)^**†**^	*p*-value	R^**2‡**^
Age	-0.26 (0.12)	**0.032**	0.0651	-0.21 (0.12)	0.088	0.0421
Sex	3.08 (15.72)	0.845	0.0005	7.71 (15.52)	0.620	0.0034
Education	-5.31 (5.26)	0.316	0.0142	-2.60 (5.31)	0.626	0.0034
Children	-0.15 (0.13)	0.262	0.0184	0.01 (0.13)	0.953	0.0001
Professional experience	-0.38 (0.11)	**0.001**	0.1423	-0.19 (0.12)	0.114	0.0352
Previous experience	-9.22 (6.03)	0.131	0.0314	-19.29 (5.68)	0.001	0.1400
Knowledge promotion	-0.08 (0.12)	0.478	0.0069	0.15 (0.11)	0.199	0.0229
Active participation	-1.47 (5.33)	0.784	0.0011	-5.21 (5.24)	0.323	0.0142

*Beta = Regression coefficient; ^†^SE = standard error of beta; ^‡^R^2^ = Coefficient of determination

Thus, the statistically significant variables entered the multivariate linear regression by the Stepwise Backward Wald method. It was verified that the “professional experience” kept a significant relation with the mean score of self-confidence post *in situ* simulation (p=0.008), therefore, the teachers who showed higher mean scores of self-confidence after educational activity had less time of professional experience. The variable “previous experience” kept significant relation with self-confidence promotion (p=0.003), which means that teachers who did not report previous experience with health complications had a greater self-confidence promotion.

## Discussion

From the characterization, we identified that 97% of the participants were female and were 39.4 years on average. A study that aimed to determine the awareness, attitudes and practices of Indian teachers on first aid supports the measure regarding female prevalence, since 82.2% of participants were women. Regarding the age of teachers, the results found in a study conducted in Korea differ from this investigation, in which the majority (34.2%) is between 20 and 29 years^15^.

Most participants (78.7%) claimed to have experienced an emergency situation throughout life, whether in the school environment or not. This datum is similar to results of other research projects, which reinforce that health complications among children in the school context are common^11,18^. Experiencing health complications may be related to the presence of children in the family nucleus, since 70% of the teachers declared having children. In a Brazilian study that assessed the knowledge of teachers and other professionals who worked in the school environment after receiving first aid training, the majority (71.4%) also reported to have already experienced a situation of complication^30^.

Among the items with lower average score of self-confidence, that one regarding the first aid to an unconscious child who is not breathing stands out, with average score of 5.53 after *in situ* simulation. According to literature, the anatomical and physiological specificities of children require a different management of the situation^31^, which may intensify the feelings of insecurity.

According to the results, the need to intervene in the face of a health complication causes a feeling of insecurity among teachers. Such result corroborates a qualitative study that aimed to understand the teachers’ role in the face of urgency and emergency situations at school^14^. According to this study, the unpreparedness, insecurity, and nervousness of the teachers for initial care of complications are common. This problematic issue is also observed in an international study^15^.

The participation in the *in situ* simulation promoted the self-confidence of participants, reiterating the relevance of courses and trainings aimed at the technical and emotional formation to manage health complications in the school environment^32^. An international investigation also attributed the teachers’ self-confidence to constant training on first aid^15^.

The results point out that teachers who had not experienced health complications throughout life had a greater score difference in the self-confidence after *in situ* simulation, which is explained by their low pre-simulation scores of self-confidence. Such result can be an indicative of the effective benefits of the educational interventions among teachers who do not have previous experiences. In this perspective, we support the assumption that repeated experiences improve self-confidence^17^. A qualitative investigation that analyzed types of knowledge and experiences of child educators on first aid reinforces the exposed by presenting the success of one of the teachers in the care of a baby with obstruction of the upper airway. According to the authors, their conduct happened due to their previous experiences^33^.

Moreover, the multivariate regression analysis pointed out that teachers with higher average scores after the *in situ* simulation were those with shorter professional experience. No study developed with teachers was found that could support this finding; however, a study that sought to associate the satisfaction with academic activities and the sociodemographic variables of 170 Nursing students revealed that younger students were more satisfied with the course and with the opportunity for development^34^.

This study assumed the *in situ* simulation while methodological strategy to reproduce the proposed educational activity and identified a significant association between the active participation in the simulated scenarios and the average score of self-confidence after *in situ* simulation, corroborating other investigations that used the simulation, which reinforce that active participation provides critical thinking and reflection, consequently expanding confidence^35^. “Making” is linked to cognitive aspects, skills and competencies for a given situation, that is, active participation is effective and promotes meaningful learning^36^. The experience of simulations proved to be positive in relation to the acquisition and increase of the level of confidence and learning^37-38^.

Self-confidence to help a child having a seizure was also strengthened after the *in situ* simulation, resembling the results of an international study that verified the effect of health education on knowledge, skills and attitudes when in the face of epilepsy, among intern teachers. According to the researchers, the teachers had limited knowledge and skills, and demonstrated negative attitudes in relation to epilepsy; however, after educational interventions on the theme, they showed significant levels of improvement regarding the attitudes and skills in managing the disease safely^19^.

It was also identified that items that obtained the lowest difference before and after educational activity mediated by the *in situ* simulation were the ones related to confidence in recognizing and managing a child with a fever, that is, teachers that already showed good self-confidence levels regarding care with fever before the activity. Such finding countered with the result found by an international study that sought to identify the knowledge, anxiety and management of fever among teachers of a daycare facility^39^, which identified high levels of anxiety when faced with a feverish child^39^.

Although the results of this study find support in literature, it is worth mentioning some limitations. The main limitation refers to the design, which did not incorporate a control group, making it impossible to identify the cause and effect relations. Another important limitation concerns the non-systematic control of the reading of the booklet among the teachers. Still, these limitations do not revoke the research results, but indicate the need for future studies with longitudinal design.

Finally, this investigation advances in the knowledge as it has the potential to subside the planning of the actions of health education in school. The identification of factors associated to self-confidence allows to propose systematized and effective educational strategies, which will certainly promote self-confidence of teachers and, consequently, a safe management of the main health complications in the school environment.

## Conclusion

In this study, we aimed to analyze the contributions of the *in situ* simulation in the self-confidence of early childhood and elementary education teachers with relation to the initial management of health complication in school, and it was concluded that the results shown met the objective and answered the research questions.

From the statistical analyses, it was observed that teachers do not feel confident to manage health complications in school, but after the *in situ* simulation, a self-confidence promotion was observed, especially among those with shorter professional experience, without previous experience with similar situations and who worked actively in the simulated scenarios.

In this way, the elaborations of educational activities that allow a greater number of active participants is relevant, respecting the desire and willingness of participants. Also, there should be new investigations to compare the promotion of self-confidence between the activities mediated by the simulation and educational activities traditionally conducted, that is, those in which the learner keeps a passive posture and the educator is responsible for the educational process, such as theoretical classes.

## References

[1] Silva JVF, Silva EC, Silva EG, Ferreira AL, Rodrigues APRA (2016). Perfil da morbidade hospitalar por doenças respiratórias na
infância de 0 a 9 anos na cidade de Maceió – AL no período de 2008 a
2014. Cad Graduação - Ciências Biológicas e da Saúde UNIT-ALAGOAS.

[2] Pina JC, Moraes SA, Freitas ICM, Mello DF (2017). Role of primary health care in child hospitalization due to
pneumonia: a case-control study. Rev. Latino-Am. Enfermagem.

[3] Malta DC, Mascarenhas MDM, Neves ACM, Silva MA (2015). Treatment of childhood injuries and violence in public emergency
services. Cad Saúde Pública.

[4] Oliveira ADS, Lopes AG, Lisboa JM, Campelo DML, Marinho CMM, Araujo ALSC (2012). Performance of teachers to children in case of accidents at
school. Rev Interdisciplinar UNINOVAFAPI.

[5] Copetti CL, Maciel GW, Daminelli CR, Gualtieri PD, Souza RL (2014). Calls a teens accident victims of household in child in the south
hospital materno Santa Catarina. Rev Inova Saúde.

[6] Masih S, Sharma RK, Kumar A (2014). Knowledge and practice of primary school teachers about first aid
management of selected minor injuries among children. Int J Med Public Health.

[7] Galindo NM, Caetano JA, Barros LM, Silva TM, Vasconcelos EMR (2017). First aid in schools: construction and validation of an
educational booklet for teachers. Acta Paul Enferm.

[8] Carvalho LS, Alarcão ALC, Barroso PD, Meireles GOAB (2014). The Approach of First Aid Performed by Teachers in a State
Educational Unit at Anapólis - GO. Ensaios Cienc Biol Agrar Saúde.

[9] Li F, Sheng X, Zhang J, Jiang F, Shen X (2014). Effects of pediatric first aid training on preschool teachers: a
longitudinal cohort study in China. BMC Pediatr.

[10] Ngayimbesha A, Hatungimana O (2015). Evaluation of first aid knowledge among elementary school teacher
in Burundi. Int J Sports Sci Fitness.

[11] Galindo NM, Pereira JC, Muniz ML, Mallmann DG, Souza NMG, Neri MFS (2016). Health Education Intervention on First Aid in School: Integrative
Review. Int Arch Med.

[12] Joseph N, Narayanan T, bin Zakaria S, Nair AV, Belayutham L, Subramanian AM (2015). Awareness, attitudes and practices of first aid among school
teachers in Mangalore, south India. J Prim Health Care.

[13] Sena SP, Ricas J, Viana MRA (2008). Perception of accidents in school by elementary level educators,
Belo Horizonte. Rev Med Minas Gerais.

[14] Rodrigues KL, Antão JYFL, Sobreira GLS, Brito RN, Freitas GLS, Serafim SC (2015). Teacher’s Knowledge about First Aid in the School Environment:
Strategies to Develop Skills. Int Arch Med.

[15] Hwang JY, Oh ES, Cho KJ (2016). A study on the self-confidence in performance and education
demand of first aid in kindergarten and daycare center
teachers. J Korea Acad Industr Cooperat Soc.

[16] Mazzo A, Martins AJC, Jorge BM, Batista RCN, Almeida RGS, Henriques FMS (2015). Validation of the self-confidence scale of nursing care in
urinary retention. Rev Latino-Am. Enfermagem.

[17] Kim SJ, Shin H, Lee J, Kang S, Bartlett R (2017). A smartphone application to educate undergraduate nursing
students about providing care for infant airway obstruction. Nurse Educ Today.

[18] Eze CN, Ebuehi OM, Brigo F, Otte WM, Igwe SC (2015). Effect of health education on trainee teachers’ knowledge,
attitudes, and first aid management of epilepsy: An interventional
study. Seizure.

[19] Martins JCA, Baptista RCN, Coutinho VRD, Mazzo A, Rodrigues MA, Mendes IAC (2014). Self-confidence for emergency intervention: adaptation and
cultural validation of the Self-confidence Scale in nursing
students. Rev. Latino-Am. Enfermagem.

[20] Oliveira SN, Prado ML, Kempfer SS (2014). Use of simulations in nursing education: an
integrative. Rev Min Enferm.

[21] Teixeira CRS, Pereira MCA, Kusumota L, Gaioso VP, Mello CL, Carvalho EC (2015). Evaluation of nursing students about learning with clinical
simulation. Rev Bras Enferm.

[22] Sorensen JL, Ostergaard D, LeBlanc V, Ottesen B, Konge L, Dieckmann P (2017). Design of simulation-based medical education and advantages and
disadvantages of in situ simulation versus off-site
simulation. BMC Med Educ.

[23] Posner GD, Clark ML, Grant VJ (2017). Simulation in the clinical setting: towards a standard
lexicon. Adv Simul.

[24] Alkhulaif A, Julie I, Barton J, Nagle E, Yao A, Clarke S (2016). Simulación in situ: ventajas, retos y obstáculos. Latin Am J Telehealth.

[25] Polit DF, Beck CT (2011). Fundamentos da Pesquisa Clínica em Enfermagem: Avaliação da evidência
para a prática de enfermagem.

[26] Zonta JB, Eduardo AH, Okido ACC (2018). Self-confidence for the initial management of health issues in
schools: construction and validation of a visual analogue
scale. Anna Nery.

[27] Chamberlain J (2015). Prebriefing in Nursing Simulation: A Concept Analysis Using
Rodger’s Methodology. Clin Simul Nurs.

[28] Jeffries PR (2007). Simulation in nursing education: from conceptualization to
evaluation.

[29] Fisher LD, Van Belle GV (1993). Biostatistics: a methodology for the health sciences.

[30] Calandrim LF, Santos AB, Oliveira LR, Massaro LG, Vedovato CA, Boaventura AP (2017). First aid at school: teacher and staff training. Rev Rene.

[31] Abrantes AWB, Coura EMG, Bezerra ALD, Assis EV, Feitosa ANA, Freitas MA (2015). Knowledge, atitudes and nursing practices on cardiorespiratory
arrest in neonatal intermediate care unit: a qualitative study in the
northeast of Brazil. J Hum Growth Dev.

[32] Silva LGS, Costa JB, Furtado LGS, Tavares JB, Costa JLD (2017). First aid and prevention of accidents in the school environment:
intervention in the educational unit. Enferm Foco.

[33] Machado ECM, Petry AR, Somavilla VEC, Hopp LS (2017). Accidents in childhood: perception and atitudes of teachers in
child education. Rev Saúde Desenvolv.

[34] Ramos AM, Barlem JG, Lunardi VL, Barlem ELD, Silveira RS, Bordignon SS (2015). Satisfaction with academic experience among undergraduate nursing
students. Texto Contexto Enferm.

[35] Costa RRO, Medeiros SM, Martins JCA, Cossi MS, Araújo MS (2017). Perception of undergraduate nursing students on realistic
simulation. Rev Cuidarte.

[36] Mazzo A, Martins JCA, Baptista RCN, Godoy S, Coutinho VRD, Seixas CA (2017). A Simulação e a Videoconferência no Ensino de
Enfermagem. Rev Grad USP.

[37] Baptista RCN, Martins JCA, Pereira MFR, Mazzo A (2014). Students’ satisfaction with simulated clinical experiences:
validation of an assessment scale. Rev Latino-Am. Enfermagem.

[38] Martins JCA, Coutinho VR, Baptista RC, Oliveira LM, Gonçalves RF, Paiva LA (2017). Impact of a simulated practice program in the construction of
self-confidence for intervention in emergencies and its association with
knowledge and performance. J Nurs Educ Pract.

[39] Park SL, Kim JS (2016). Factors affecting daycare center teachers’ management of
childhood fever. Child Health Nurs Res.

